# Interpretable deep multimodal-based tomato disease diagnosis and severity estimation

**DOI:** 10.1038/s41598-025-21611-4

**Published:** 2025-10-29

**Authors:** Nimra Nasir, Shabana Ramzan, Basharat Ali, Sohail Jabbar, Ali Raza, Changgyun Kim, Muhammad Syafrudin, Norma Latif Fitriyani

**Affiliations:** 1 Department of CS & IT, Govt Sadiq College Women University, Bahawalpur, Pakistan; 2 Agronomic Research Station, Bahawalpur, Pakistan; 3 College of Computer and Information Sciences, Imam Mohammad Ibn Saud Islamic University (IMSIU), 11432 Riyadh, Saudi Arabia; 4 Department of Precision Medicine, Sungkyunkwan University School of Medicine, Suwon, 16419 Republic of Korea; 5 Department of Electronic and AI System Engineering, Kangwon National University, Samcheok, 25913 Republic of Korea; 6 Department of Artificial Intelligence and Data Science, Sejong University, Seoul, 05006 Republic of Korea

**Keywords:** Computational biology and bioinformatics, Mathematics and computing

## Abstract

Plant diseases pose a significant threat to global food security, particularly in regions that rely heavily on crops that are vulnerable to disease, such as tomatoes. This research addresses the inefficiencies of traditional farming solutions by presenting a novel multimodal deep learning algorithm. The algorithm leverages EfficientNetB0 for image-based disease classification and utilizes Recurrent Neural Networks (RNN) to predict disease severity based on environmental data. By integrating visual and climatological inputs, our model addresses the limitations of unimodal systems, enhancing classification accuracy and interpretability. The model achieved a disease classification accuracy of 96.40% and a severity prediction accuracy of 99.20%. Additionally, the use of LIME and SHAP explainable AI techniques improves the understanding of disease severity classification outcomes. The contributions of this study align with precision agriculture practices and advance the resilience of local food systems, particularly in economies heavily dependent on tomato production. The proposed approach has the potential to mitigate the impacts of plant diseases and enhance food security by utilizing innovative technological solutions.

## Introduction

Plant diseases pose a significant challenge to feeding the world, particularly in countries that heavily rely on crops like tomatoes, which are prone to disease. Since tomatoes are a staple ingredient in many dishes and a significant source of nutrients, they play a central role in family consumption and agriculture. With the global population continuing to grow, the demand on agricultural systems to produce more food is higher than ever before. This is in addition to the pressure exerted by the development and spread of plant pathogens, which have themselves become more frequent and intense with increasingly changing environmental conditions^1^. Among these dangers, fungal and oomycete diseases are particularly prominent due to their increasing frequency and the significant economic losses they cause, resulting in substantial yield losses of tomatoes and other high-value crops^2^. Addressing food security in the face of such threats requires innovative and targeted strategies in disease surveillance and prevention.

In the Pakistani context, tomatoes are one of the most popular vegetables and the staple ingredient in everyday meals within urban and rural communities. The country’s varied agro-climatic conditions–specifically in Punjab, Sindh, and Balochistan—enable year-round production, making Pakistan one of the prominent tomato-producing countries in South Asia. The per capita consumption of tomatoes in Pakistan is estimated to be around 8 to 10 kilograms per annum, used as a staple in curries, gravies, salads, chutneys, and processed foods such as ketchup. Being a high-demand crop, any change in production, usually due to disease outbreaks, is directly reflected in price inflation and disruption of the supply chain. This is immediately felt economically by both producers and consumers. Successful prediction and control of disease are therefore essential, not only to safeguard the health of crops but also to provide affordability and access to tomatoes as part of the daily diet of millions.

The need for precise and timely prediction of the onset and intensity of plant disease is further compounded by the impacts of climate change. Increased global temperatures, altered rainfall patterns, and extreme weather conditions have created environments that are increasingly conducive to the growth and transmission of plant pathogens^3–5^. Not only do the environmental changes complicate disease detection, but they also compromise plant immune systems, lowering their natural defenses. In addition, the complex interactions between plants and their environmental microbiomes are frequently disrupted by such stressors, compromising the plant’s resilience and its capacity to fight infection^6,7^. Conventional farming practices and abatement strategies are insufficient on their own these days and need to be supplemented by innovative, adaptive technologies.

Recent research in deep learning and artificial intelligence has had a profound impact on the field of plant disease identification and prediction. Convolutional Neural Networks (CNNs), such as EfficientNet and ResNet, have demonstrated high accuracy in classifying image-based tasks, particularly in detecting diseases in plant leaves. Concurrently, time-series models such as Long Short-Term Memory (LSTM) networks have proven effective in predicting the severity of the disease by examining environmental factors, including humidity, temperature, and rainfall. Perhaps most significant, however, are new multimodal models that incorporate various data streams—such as leaf imagery and current weather conditions—offering significant potential for comprehensive agricultural monitoring.

While previous studies have utilized CNNs for image classification^8^ or LSTMs for severity prediction^7^, our work presents a novel, interpretable multimodal framework that uniquely integrates a vision-based classifier (EfficientNetB0) with a weather-based severity predictor (MLP) under a unified, explainable AI (XAI) framework. To the best of our knowledge, this is the first study to employ a late-fusion strategy of this kind for tomato disease diagnosis, complemented by both LIME (for image modality) and SHAP (for weather modality) to provide comprehensive model interpretability. This approach directly addresses the limitations of single-modality systems and the ‘black-box’ nature of previous deep learning models in agriculture^9,10^.

### Study contributions

In this work,

- We introduce a new multimodal deep learning method for detecting and estimating the severity of tomato disease using the PlantVillage dataset.
- We use EfficientNetB0, accompanied by Local Interpretable Model-agnostic Explanations (LIME) for added interpretability of prediction.
- To estimate severity, we leverage an RNN alongside SHapley Additive exPlanations (SHAP).
- Our solution corrects these shortcomings by incorporating understandable, multimodal analysis for more resilient plant disease management.

The rest of the paper is structured as follows: “Literature analysis” gives the literature review of tomato disease diagnosis and prediction of severity. “Proposed methodology” describes our proposed multimodal deep learning approach, utilising EfficientNetB0 and RNN models, along with LIME and SHAP explanations. “Results and discussions” provides the experimental results, performance analysis, and interpretability findings. “Conclusion” describes the limitations and research directions.

## Literature analysis

Several research studies have investigated environmental dynamics and the transmission of plant diseases. Miedaner and Garbelotto^3^ emphasised the importance of human-mediated plant migration in the transmission of pathogens, highlighting the need for proactive disease monitoring systems. Rigg et al.^4^ investigated the vulnerability of alpine plant species to Phytophthora pathogens, suggesting that species-specific susceptibility needs to be considered in detection models. Thompson et al.^5^ examined the interactive effect of rainfall and temperature variation on disease incidence, demonstrating how climate variability complicates the prediction of pathogens. Cheng et al.^6^ demonstrated how environmental stress affects plant–microbe interactions, emphasizing the ecological context as a key factor in modeling plant health. Trivedi et al.^11^ presented the role of microbial communities in plant resilience, thereby reinforcing the need to incorporate biological and environmental information into disease analysis.

Desaint et al.^8^ demonstrated that stress caused by heat increases plant susceptibility to pathogens, thus necessitating real-time environmental monitoring for predictive systems. Based on this, Trivedi et al.^7^ analyzed the transformed function of plant–microbiome interactions in the face of climate change, calling for adaptive modeling approaches in precision agriculture. Kamilaris and Prenafeta-Boldú^9^ reviewed deep learning in agriculture, naming CNNs and RNNs as the basis models for plant disease detection and prediction. Zhang et al.^10^ proposed Vision Transformers for plant disease classification, showing greater performance compared to conventional CNNs in image classification. Nagamani and Sarojadevi^12^ applied deep learning to images of tomato leaves with high accuracy in disease classification, thereby proving the merit of CNNs in tomato-specific research.

Xu et al.^13^ applied LSTM networks to forecast disease severity from weather time series data, underscoring the temporal dependency of climate and disease outbreaks. Saleh et al.^14^ proposed a transformer multimodal model that integrates image and climate data, demonstrating the capability of heterogeneous sources for early disease detection. The PlantVillage dataset^15^ has been used to train strong classification models with extensive labelled images of leaf diseases, particularly for tomato. Liu et al.^16^ proposed the Swin Transformer, a computationally effective vision model that processes image patches hierarchically, enhancing scalability and accuracy in plant image analysis. Yin et al.^17^ introduced A-ViT, which enhances Vision Transformer performance by incorporating adaptive tokens, making it applicable to low-resource agricultural tasks.

Also, recent studies by Kunduracioglu et al.^18,19^ have demonstrated the effectiveness of various deep learning architectures, including CNNs, ResNet, and EfficientNet, for disease detection across multiple crops, including apples, tomatoes, sugarcane, and grapes, further validating the application of these architectures in agricultural disease diagnosis.

Liu et al.^20^ utilized ResNet-18 for medical image diagnosis, demonstrating its transferability to other applications, such as plant pathology, due to its ability to extract profound features. Almohimeed et al.^21^ integrated ensemble learning with SHAP-based explanations to enhance model transparency in disease prediction, offering a framework that aligns with explainability objectives in agriculture. Finally, Cardoso et al.^22^ created MONAI, an open-source deep learning platform for medical imaging, which can be ported to organize and standardize AI workflows in agriculture.

Recent research continues to advance the field with novel architectures and approaches. For instance, Upadhyay and Gupta^23^ demonstrated a modified ResNeXt model for detecting multi-crop diseases in heterogeneous datasets, highlighting the need for robust feature extraction. Further extending this work, SegLearner^24^ presents a segmentation-based methodology for predicting disease severity, which aligns with our objective of moving beyond simple classification. Beyond disease focus, similar feature fusion techniques have also been effectively applied to challenges like semantic weed detection, as seen in the 3SW-Net architecture^25^, underscoring the versatility of deep learning solutions in precision agriculture.

### Research gap analysis

There have been a few attempts to integrate image-based classification with real-time numeric forecasting of disease severity, particularly within an explainable AI (XAI) framework.

- Single-modal datasets like images or environmental information alone are used for existing models of tomato disease diagnosis, which restricts accuracy and robustness^12,13^.
- Most deep learning models applied in agriculture are not explainable, which renders them less reliable for real-world applications^14,21^.
- Little work integrates CNN-based image classifiers and RNN-based severity predictors into a modular and interpretable multimodal framework^13,14^.
- Previous attempts with LIME or SHAP have not been extended extensively to agriculture-specific settings for both disease classification and severity estimation at once^21^. Our major contribution in this work is
- Designed a new multimodal architecture that leverages image-based classification (EfficientNetB0 + LIME) and severity estimation from environmental data (RNN + SHAP).
- Presented a late fusion strategy for fusing predictions from CNN and RNN models into one interpretable decision output, both improving accuracy and interpretability.
- Utilized LIME and SHAP to enhance explainability, ensuring the system was transparent and usable by agricultural decision-makers^21^.
- Created top performance in classification (96.7%) and severity prediction (99.2%), outperforming current state-of-the-art models^9,10,14^.

Table 1 summarizes key studies referenced in this chapter, highlighting the authors, methodologies, datasets used, and primary contributions.

**Table 1:** The summary of existing literature.

Ref	Technique used	Limitation	Future work
^12^	CNN-based deep learning for tomato leaf disease classification	Focused only on image classification; lacks severity prediction and interpretability	Add severity prediction and integrate explainable AI like LIME
^13^	LSTM for predicting crop disease severity using weather time series	Lacks integration with image data; single-modality	Combine with CNN image classifiers for multimodal prediction
^14^	Transformer-based multimodal model combining image and climate data	Complex architecture; limited real-time transparency	Enhance with SHAP/LIME and optimize for real-time agricultural use
^15^	PlantVillage dataset of labeled plant leaf images (including tomato)	Image-only data; lacks weather or time-series annotations	Extend with synthetic or real numeric datasets for severity modeling
^21^	SHAP-based explainable AI ensemble for disease detection	Applied in healthcare, not directly tested on plant data	Apply SHAP to agricultural RNN/CNN outputs for interpretable predictions

## Proposed methodology

**Fig. 1:**
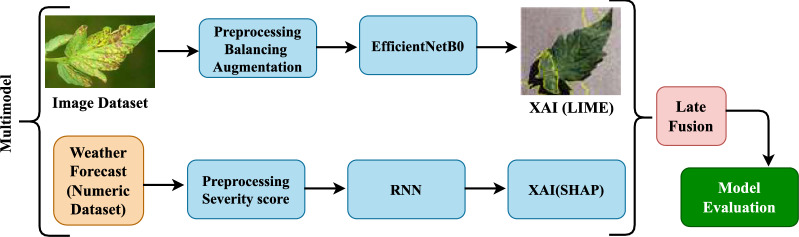
The methodology framework for the proposed system.

This section presents the framework of deep learning multimodal for tomato leaf disease detection and estimation of disease severity by fusing image and weather-based data. Our model consists of two separate, independently trained models: an EfficientNetB0-based classifier for disease type identification from leaf images, and an RNN that estimates the severity of the identified disease based on a sequence of weather observations. To make our models interpretable and reliable, we utilise LIME and SHAP for post-hoc explanation. The predictions from both models are combined through a late fusion approach to obtain a single, interpretable, and actionable decision. The architectural framework is shown in Fig. 1.

### Data collection

Firstly, we employed two complementary datasets to facilitate disease classification and severity prediction tasks. The first is the publicly available PlantVillage dataset^15^, which includes a subset of thousands of labelled tomato leaf images spanning multiple disease classes. For the experiments, we selected the classes listed in Table 2.

**Table 2:** The distribution of tomato leaf disease image classes.

Class name	Number of image files
Tomato___Bacterial_spot	1702
Tomato___Early_blight	800
Tomato___Late_blight	1527
Tomato___Leaf_Mold	761
Tomato___Septoria_leaf_spot	1417
Tomato___Spider_mites Two-spotted_spider_mite	1341
Tomato___Target_Spot	1123
Tomato___Tomato_Yellow_Leaf_Curl_Virus	4286
Tomato___Tomato_mosaic_virus	299
Tomato___healthy	1273
Total	14,529

The second set of data^26^ comprises numeric daily weather records, including temperature (${}^{\circ }$C), humidity (%), rain (yes/no), and wind speed (km/h), as shown in Table 3. These environmental factors have been proven to influence the development and severity of plant diseases, and are utilized in predictive models to forecast disease severity levels.

**Table 3:** The weather forecast dataset used for disease severity prediction.

Feature name	Description
Temperature	Air temperature in Celsius
Humidity	Relative humidity in percentage
Wind_Speed	Wind speed in km/h
Cloud_Cover	Percentage of sky covered with clouds
Pressure	Atmospheric pressure in h(Pa)
Rain	Rainfall status (categorical: ’rain’ or ’no rain’)

### Data preprocessing

Image dataset: after selecting the dataset, the next step is to preprocess the data for further use. Firstly, all images of tomato leaves were resized to $224 \times 224$ pixels to align with the input size accepted by EfficientNetB0. The images were then converted to grayscale and subsequently expanded to 3-channel RGB format to be compatible with the input layers of CNNs. A yellow color filter was applied by amplifying the red and green channels to highlight areas infected with disease. Lastly, pixel values were clipped and normalized to the range [0, 1] to stabilize training and enhance model convergence.

Numeric dataset: the weather dataset with available features, temperature, humidity, wind speed, cloud cover, pressure, and precipitation, was first loaded. The categorical feature rain was encoded in binary form to allow for numerical manipulation.

A custom disease severity score was created by weighting the meteorological variables to reflect their impact on disease development. The score was computed as a weighted sum of the normalized weather variables:

1 $$\begin{aligned} \text {Severity Score} = 0.2T + 0.25H + 0.15W + 0.1C + 0.2P + 0.1R \end{aligned}$$

where *T* is the temperature, *H* is the humidity, *W* is the wind speed, *C* is the cloud cover, *P* is the pressure, *R* is the rainfall.

The continuous severity score was divided into three disease severity classes:

- Low: Score $$\le 0.4$$
- Medium: $$0.4 < \text {Score} \le 0.7$$
- High: Score $$> 0.7$$

The disease severity score was normalized using Min-Max scaling to bring all values into the [0, 1] range. These normalized scores were categorized into the three severity classes above. Weather feature variables were also scaled using Min–Max normalization and reshaped into the required 3D input format for RNNs. The class labels were one-hot encoded for multi-class classification as follows:

2 $$\begin{aligned} \text {Label (one-hot)} \in \{[1,0,0], [0,1,0], [0,0,1]\} \end{aligned}$$

These steps completed the preprocessing phase, ensuring that the input data was clean, normalized, and correctly formatted for training.

Severity label generation: the original PlantVillage dataset provides disease classes but not severity labels. To create a valid target variable for severity prediction, we established a severity mapping based on agronomic literature^27^. Each disease class was assigned a severity level (Low, Medium, High) reflecting its potential impact on yield and plant health. For instance, *healthy* plants are assigned ‘Low‘ severity, while diseases known to cause rapid plant destruction, like *Late Blight*, are assigned ‘High‘ severity. This expert-informed mapping provides a biologically grounded target for the severity prediction model, moving beyond arbitrary synthetic scores. The full class-to-severity mapping is detailed in Table 4

**Table 4:** Mapping of tomato disease classes to expert-defined severity levels.

Disease class	Severity level
Tomato healthy	Low
Tomato early blight	Medium
Tomato late blight	High
Tomato leaf mold	Medium

### Data balancing

After preprocessing, data balancing was performed on the image dataset. A combination of random undersampling and oversampling via image duplication was used. Some classes had over 3000 images while others had fewer than 500. A Python script randomly deleted excess images from overrepresented classes (undersampling) and duplicated images from underrepresented classes (oversampling) until all classes contained exactly 500 images. Unique filenames were assigned to duplicated images to avoid conflicts. This process ensured equal class representation, essential for training a balanced and unbiased deep learning model.

### Image augmentation

To enhance model generalization and simulate the natural variability among leaf appearances, image augmentation techniques were applied during training. These included:

- Random rotation ($$\pm 20^\circ$$)
- Horizontal and vertical flipping
- Zooming
- Shifting
- Brightness adjustment
- Shearing

These transformations were dynamically applied using Keras’ ImageDataGenerator, which increased the diversity of the training set and reduced overfitting.

### Applied learning models

#### Disease classification using EfficientNetB0

**Fig. 2:**
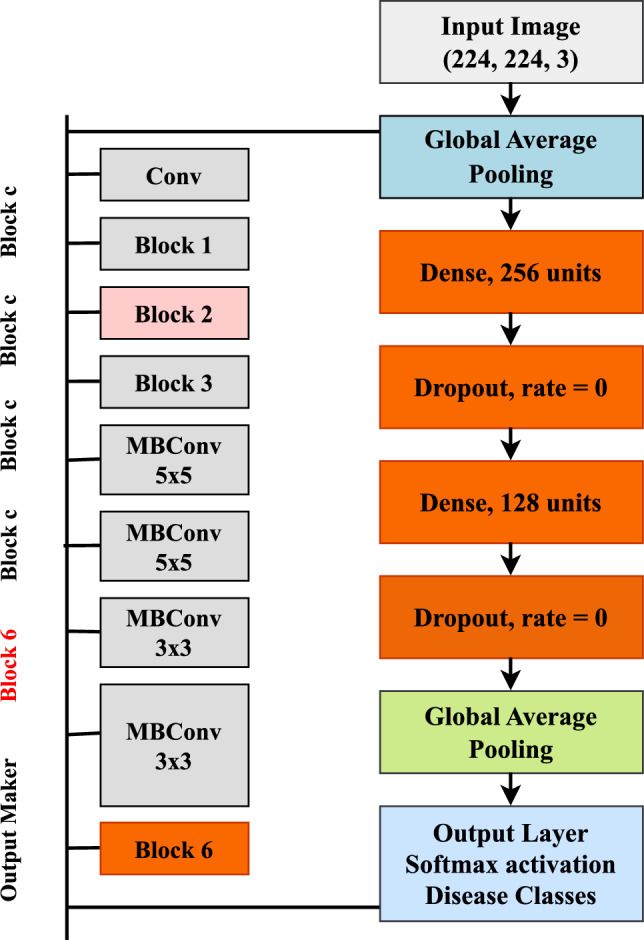
The architecture of the EfficientNetB0 model used for tomato leaf disease classification.

We used EfficientNetB0 for the image classification task, as it is computationally efficient and achieves good accuracy for small datasets^28,29^. We designed a deep learning model based on EfficientNetB0 as the starting point for classifying tomato leaf diseases. EfficientNet is a convolutional neural network whose width, depth, and resolution are scaled through a compound factor, and it is efficient and accurate with fewer parameters^30^. The architecture of the EfficientNetB0 layers is shown in Fig. 2.

Input and feature extraction (EfficientNetB0 Base): the model takes input images that are formatted as (224, 224, 3) for RGB tomato leaf images. The input is forwarded through the EfficientNetB0 model, which is frozen except for the top 10 layers. EfficientNetB0 is a convolutional neural network comprising various deep blocks of depthwise separable convolutional layers, squeeze-and-excitation (SE) blocks, and swish activation functions. Each convolution operation in the convolutional blocks uses learnable filters across space to extract local and global texture patterns relevant for disease symptoms.

In the case of an image *X*, the convolutional operation with an arbitrary kernel of some size *K* is defined by Eq. (3):

3 $$\begin{aligned} F_{i,j,k} = \sum _{m,n} \left( X_{i+m, j+n} \cdot K_{m,n,k} \right) + b_k \end{aligned}$$

where $F_{i,j,k}$ is the output feature map computed at position (*i*, *j*) for the *k*-th filter, and $b_k$ is the bias term. The feature extraction process is carried out through various layers, enabling the model to extract hierarchical features from the image.

To fine-tune the model for the tomato leaf dataset, the EfficientNetB0 network was frozen except for the top 10 layers, which were allowed to train and learn more specific visual features, building on general features extracted from the base ImageNet dataset.

Global average pooling: after feature extraction, the data is passed through a GlobalAveragePooling2D layer. This operation condenses each feature map into a single representative value by averaging across all spatial coordinates. The result is a reduced feature set that emphasizes the most salient global characteristics.

Dense and dropout layers: the combined features are passed through a dense layer with 256 units, followed by a dropout layer with a rate of 0.3 for regularization against overfitting. This is followed by a second dense layer with 128 units and a dropout rate of 0.2. These dense layers help the model to learn complex relationships between the extracted visual features and the corresponding disease classes.

Output layer: the final layer is a dense layer with softmax activation and one output unit per disease class. It returns a probability distribution over all classes, and the class with the highest probability is selected as the prediction. This structure supports multi-class classification of tomato leaf diseases.

Model compilation parameters: the model is compiled using the Adam optimizer with a learning rate of $2 \times 10^{-4}$, the categorical cross-entropy loss function, and accuracy as the performance metric. This parameter configuration, as shown in Table 5, enables robust and efficient training for multi-class classification tasks.

**Table 5:** The key hyperparameters used for training the EfficientNetB0-based tomato leaf disease classification model.

Hyperparameter	Value	Description
Optimizer	Adam(learning rate= 2e-4)	Adaptive optimizer for faster convergence
Loss Function	categorical crossentropy	Suitable for multi-class classification
Metric	Accuracy	Model performance metric
Epochs	10	Number of complete passes over the training data
Batch Size	32	Number of images processed in each training/validation step

### Severity prediction using RNN on weather data

**Fig. 3:**
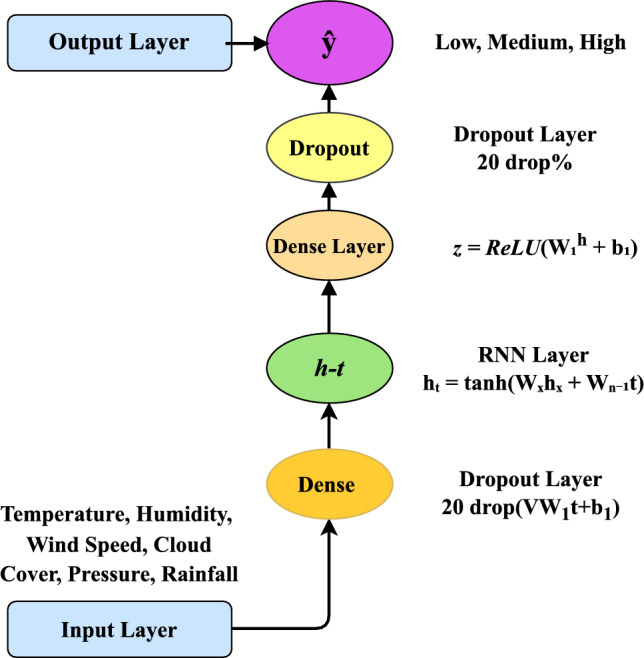
The architecture of the RNN model used for weather-based disease severity prediction.

We developed an RNN to predict the severity of tomato plant diseases based on historical weather data. The dense layers work as a Multi-Layer Perceptron (MLP) for classification. The architecture of the RNN model is shown in Fig. 3.

Input layer: the model begins by receiving a weather feature vector consisting of six parameters: temperature, humidity, wind speed, cloud cover, pressure, and rainfall. These features are reshaped into a sequence format with a shape of (6, 1), where each weather attribute is treated as a separate time step. This structure allows the RNN^31,32^ to process the input sequentially, even though the data is not time-series in nature.

RNN layer: the reshaped input is passed to an RNN layer consisting of 64 units and using the tanh activation function. This layer is responsible for capturing internal relationships among the input features by maintaining and updating a hidden state. The hidden state at each time step $h_t$ is calculated using Eq. (4):

4 $$\begin{aligned} h_t = \tanh (W_{xh} x_t + W_{hh} h_{t-1} + b_h) \end{aligned}$$

Here, $x_t$ represents the input at time *t*, $h_{t-1}$ is the hidden state from the previous step, $W_{xh}$ and $W_{hh}$ are the input and recurrent weight matrices, respectively, and $b_h$ is the bias term. The $\tanh$ activation introduces non-linearity, enabling the model to learn more complex patterns.

Dropout layer: the output of the RNN is then passed through a Dropout layer with a dropout rate of 0.2. This regularization technique helps prevent overfitting by randomly deactivating 20% of the neurons during training. Mathematically, the dropout operation modifies the hidden state as shown in Eq. (5):

5 $$\begin{aligned} \text {Dropout}(h_t) = {\left\{ \begin{array}{ll} 0 & \text {with probability } p \\ \frac{h_t}{1-p} & \text {with probability } 1-p \end{array}\right. } \quad \text {where } p = 0.2 \end{aligned}$$

By scaling the retained outputs by $\frac{1}{1-p}$, the overall expected activation remains consistent during training.

Dense Layer: the regularized output is then fed into a Dense (fully connected) layer of MLP with 32 neurons and a ReLU activation function. This layer projects the learned representations from the RNN into a higher-dimensional feature space. The transformation is computed as in Equation (6):

6 $$\begin{aligned} z = \text {ReLU}(W_1 h_t + b_1) \end{aligned}$$

Here, $W_1$ is the weight matrix, $b_1$ is the bias, and *z* is the resulting activation. The ReLU function introduces sparsity and improves learning efficiency by zeroing out negative values.

Output layer: the model’s final output is directed through a Dense layer comprising three neurons, each representing one of the severity categories: Low, Medium, and High. This layer implements the softmax activation function, which transforms the raw output values into probabilities, as computed in Equation (7):

7 $$\begin{aligned} y_i = \frac{e^{z_i}}{\sum _{j=1}^{3} e^{z_j}} \end{aligned}$$

Here, $z_i$ is the logit for class *i*, and the denominator ensures that the output probabilities across all classes sum to one. The model then designates the class with the highest probability as the predicted severity level.

Model training parameters: the subsequent configuration defines the hyperparameters listed in Table 6, which are utilized to train the Sequential RNN model for disease severity classification using weather data. The settings were chosen judiciously to maximize model performance without overfitting and ensure effective training.

**Table 6:** The hyperparameter settings for the RNN-based weather severity classification model.

Hyperparameter	Value	Description
Loss Function	categorical crossentropy	Suitable for multi-class classification with one-hot encoded targets
Optimizer	Adam	Adaptive learning optimizer
Metric	Accuracy	Used to monitor classification performance
Epochs	50	Number of complete training passes
Batch Size	32	Number of samples processed at once

### eXplainable Artificial Intelligence (XAI)

We trained this model for 25 epochs using the RMSProp optimizer and the categorical cross-entropy loss function. We used SHAP to make the predictions interpretable. SHAP allows us to understand how much each predictor (such as temperature, humidity, or rainfall) influences the model’s prediction. It also informs us about factors associated with an increase or decrease in disease severity concerning specific weather metrics. This pandemic (and our models), therefore, becomes much more interpretable and trustworthy.

SHAP works by assigning each input feature a “value” that collectively contributes to the model output for classifications. In this case, we applied SHAP to examine which weather features and on what days had the most significant effect on the predicted severity level. For instance, the predictor could express that several days of sustained high humidity increased the risk of severe disease to a relatively higher degree. This insight enables agri-experts to explain weather features in terms of severity prediction, while also providing reasonable accuracy in predicting severe disease.

#### LIME explainability

To enhance interpretability, the Local Interpretable Model-agnostic Explanations (LIME) framework is employed. LIME elucidates the model’s predictions by systematically perturbing input data–such as masking different superpixels in an image–and observing the effects on the output. In this context, LIME is used to explain the model’s classification decisions regarding specific test samples. It constructs a local surrogate model and generates heat maps to visually indicate which regions of the leaf image have the most significant influence on the prediction. This approach enables verification that the model’s decisions align with biologically plausible features, such as visible disease lesions, thus fostering confidence in the model’s diagnostic reliability.

### Decision-level late fusion

After training, the two models operate independently of each other. The EfficientNetB0 model processes an input image to predict a disease class such as late blight. Simultaneously, the MLP model processes the current weather feature vector to predict a severity level such as High. The final output is a straightforward concatenation of these two predictions, providing a comprehensive diagnosis such as Predicted Disease: Late Blight Severity: High. This modular, late-fusion approach ensures that each model excels at its specialized task, allowing for the independent improvement or replacement of any module in the future.

### Model evaluation

We compared the models based on performance metrics. Accuracy, precision, recall, F1-score, and confusion matrices were computed for the disease classification model. Accuracy was calculated, and class-wise performance was computed for the severity prediction model. We also performed qualitative analysis of the LIME heatmaps and SHAP plots to determine the interpretability of the models. These analyses ensure that our system not only functions effectively but also delivers transparent and actionable outputs for real-world agricultural decisions.

## Results and discussions

In this section, we present the results of our image-based EfficientNet model and weather-based RNN model for predicting the disease and severity of tomato leaves. We evaluate the model’s performance using accuracy, classification reports, and confusion matrices. We interpret the results in light of their applicability, credibility, and usability in real agricultural settings.

### Data preprocessing outcomes

**Fig. 4:**
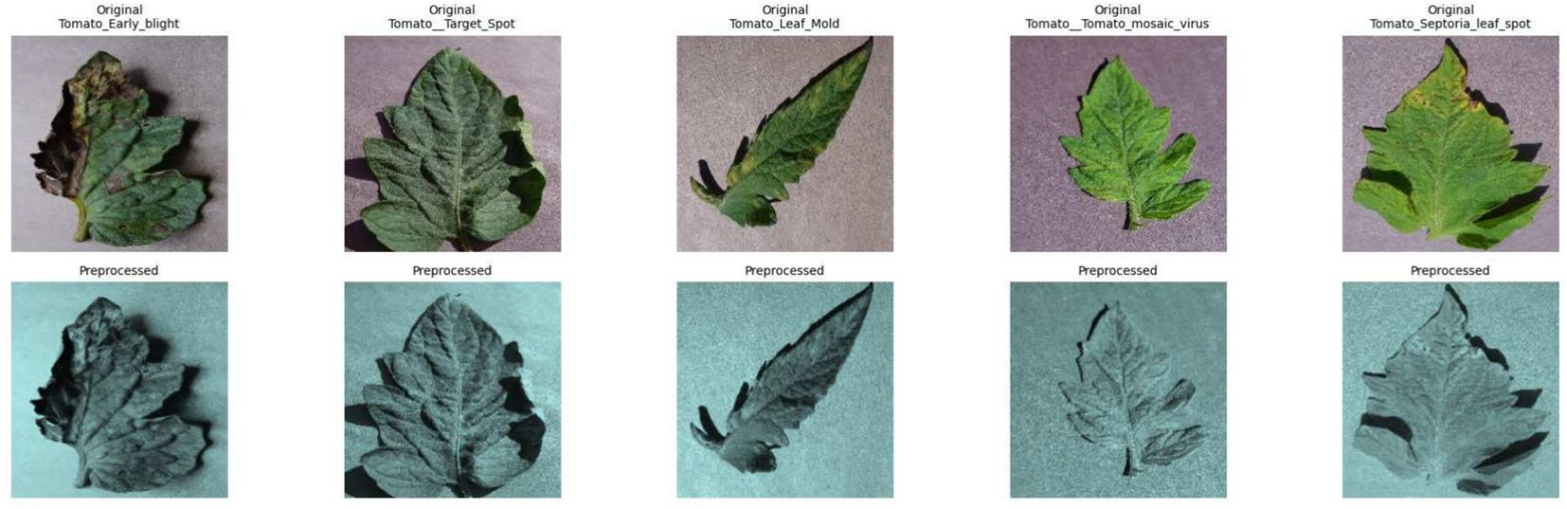
The original and preprocessed image samples used in the EfficientNetB0 model pipeline.

Preprocessing involved resizing all the images of tomato leaves to 224$\times$224 pixels, yellowish tint, and normalizing them to the [0,1] range. Normalization was performed to enable the EfficientNetB0 input layer to utilize them and enhance model convergence. Figure 4 shows the original and preprocessed images.

### Data balancing results

**Fig. 5:**
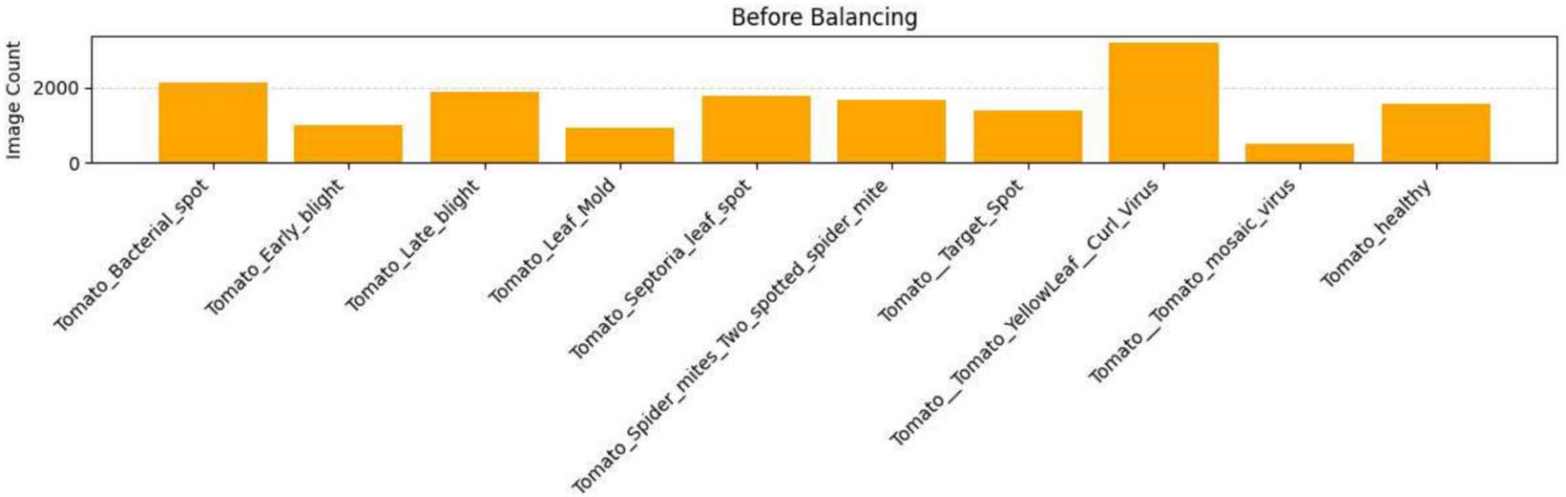
The bar chart showing image count per class before dataset balancing.

**Fig. 6:**
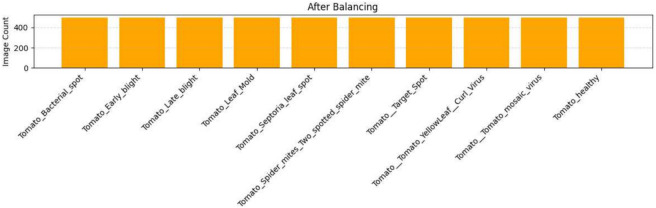
The bar chart of image count per class after balancing (each class = 500 images).

The original data set suffered from severe class imbalance: for example, some classes had over 4000 instances, while others had fewer than 300. The original dataset classes are shown in Fig. 5. Such an imbalance may skew the classifier toward the majority classes. The balanced classes are shown in Fig. 6. To counteract this, we employed a hybrid balance strategy:

- Oversampling: The minority classes were oversampled to 500 images.
- Undersampling: Randomly down-sampled the overrepresented classes to 500 images.

This balancing ensured the equitable representation of classes, allowing the classifier to learn the same number of features from each disease and thereby improve generalization.

### Image augmentation outcomes

To simulate natural variation on leaves and achieve maximum generalizability, the following augmentation techniques were employed using ImageDataGenerator:

- Random rotation ($$\pm 20^{\circ }$$)
- Horizontal & vertical flips
- Zooming, brightness shift
- Shearing and translation

**Fig. 7:**
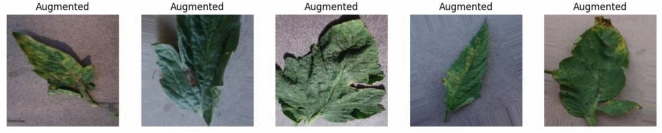
The augmented versions of tomato leaf images used to enhance dataset diversity.

These were utilized on the fly during training, and in effect, the diversity of the dataset was effectively doubled. The augmented images are shown in Figure 7. This step reduced overfitting and improved the model’s robustness to diverse lighting, orientation, and background conditions commonly encountered in field environments.

### Tomato disease classification results

**Fig. 8:**
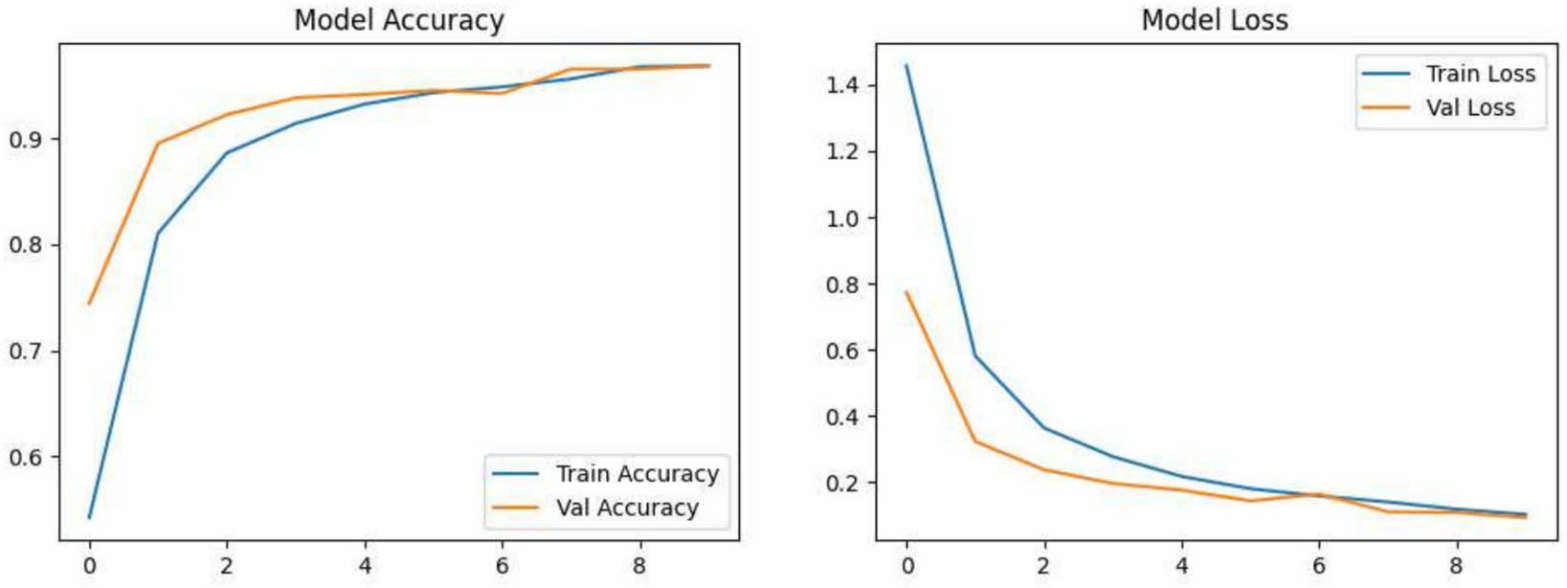
The training curves analysis.

The EfficientNetB0 model proposed was trained on an augmented, balanced dataset of tomato leaf diseases, achieving an overall classification accuracy of 96.40%. Class-wise performance analysis reveals decent precision and recall across all categories. However, lower per-class F1-scores in a few instances suggest difficulties with visual similarity among certain diseases, as shown in Table 7. The training results are illustrated in Fig. 8.

**Table 7:** The classification report of the EfficientNetB0 model for tomato leaf disease identification.

Class name	Precision	Recall	F1-score
Tomato bacterial spot	1.00	0.96	0.98
Tomato early blight	0.91	0.94	0.93
Tomato late blight	0.98	0.95	0.96
Tomato leaf mold	0.97	0.99	0.98
Tomato septoria leaf spot	0.97	0.97	0.97
Tomato spider mites (two-spotted spider mite)	0.93	0.99	0.96
Tomato target spot	0.97	0.93	0.95
Tomato yellow leaf curl virus	0.99	0.99	0.99
Tomato mosaic virus	1.00	1.00	1.00
Healthy tomato	1.00	1.00	1.00

**Fig. 9:**
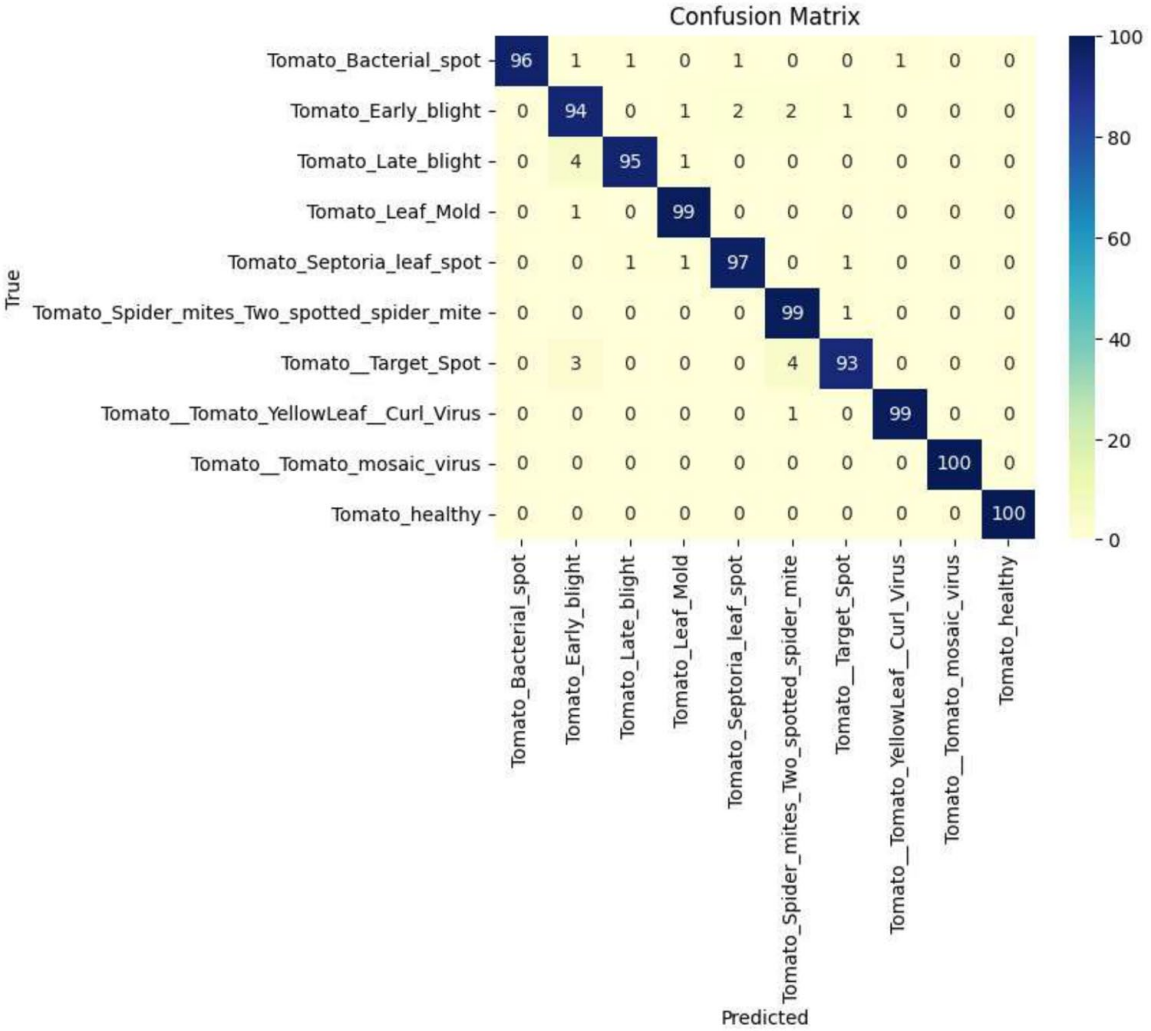
The confusion matrix illustrating the classification performance of the EfficientNetB0 model.

The confusion matrix in Fig. 9 expresses the prevalence of correct classifications but also reveals small misclassifications between closely related disease classes, indicating the intricacy involved in fine-grained disease identification.

### LIME-based explainability for image modality

**Fig. 10:**
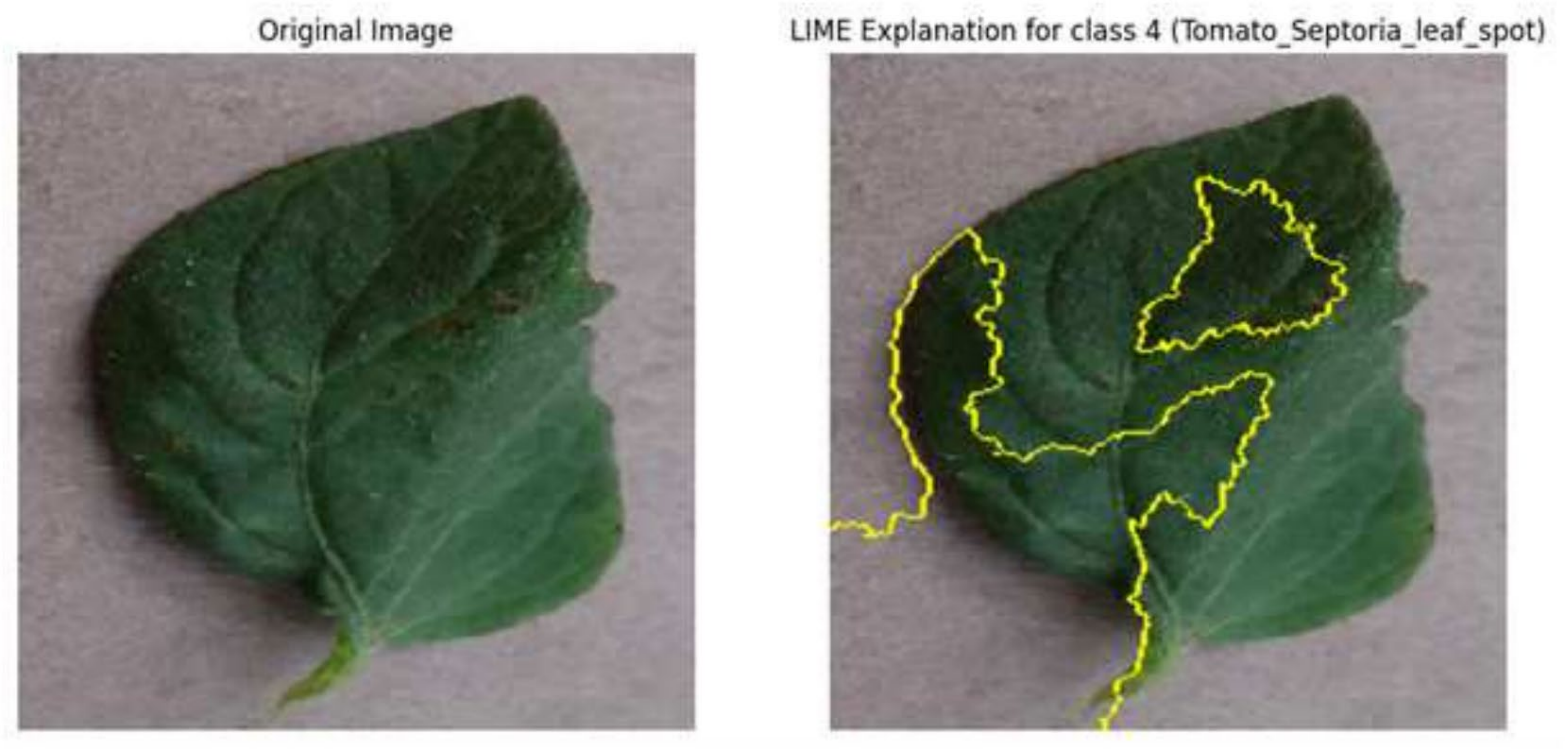
The LIME-based explainability visualization highlighting key features influencing model predictions.

We utilized LIME visualizations over the EfficientNetB0 model to enhance interpretability by highlighting the essential image areas that influence predictions. The findings validate that the model focuses on symptomatic parts of leaves, such as lesions, discoloration, and deformations, which align with recognized agronomic signs of disease. This alignment enhances the model’s trustworthiness for real-world agricultural surveillance, as illustrated in Figure 10. These results demonstrate the model’s strong discriminative power and reliability in identifying even subtle disease patterns.

### Severity prediction from weather data

The RNN model, trained on 7-day-long meteorological sequences of temperature, humidity, and rainfall, achieved 99.20% accuracy in classifying the severity of the disease into Low, Medium, and High categories. The classification report for the RNN model in predicting weather-based disease severity is presented in Table 8.

**Table 8:** The classification report of the RNN model for weather-based disease severity prediction.

Class	Precision	Recall	F1-score
Low	0.98	1.00	0.99
Medium	1.00	0.99	0.99
High	0.98	1.00	0.99
Macro Avg	0.99	1.00	0.99
Weighted Avg	0.99	0.99	0.99
Accuracy	0.99		

**Fig. 11:**
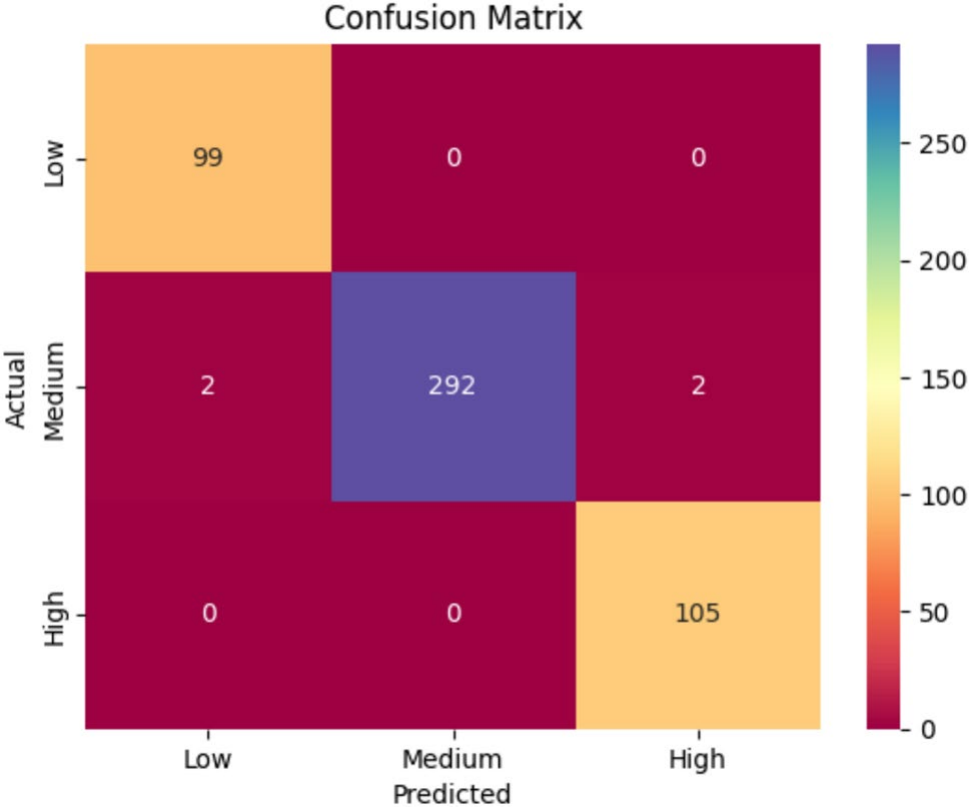
The confusion matrix depicting the classification performance of the RNN model.

The class-wise accuracy was greater than 0.98 for all levels of severity, and the confusion matrix reflects high class separability, indicating the model’s reliability in predicting severity based on weather, as shown in Fig. 11.

### SHAP explainability for weather-based severity prediction

**Fig. 12:**
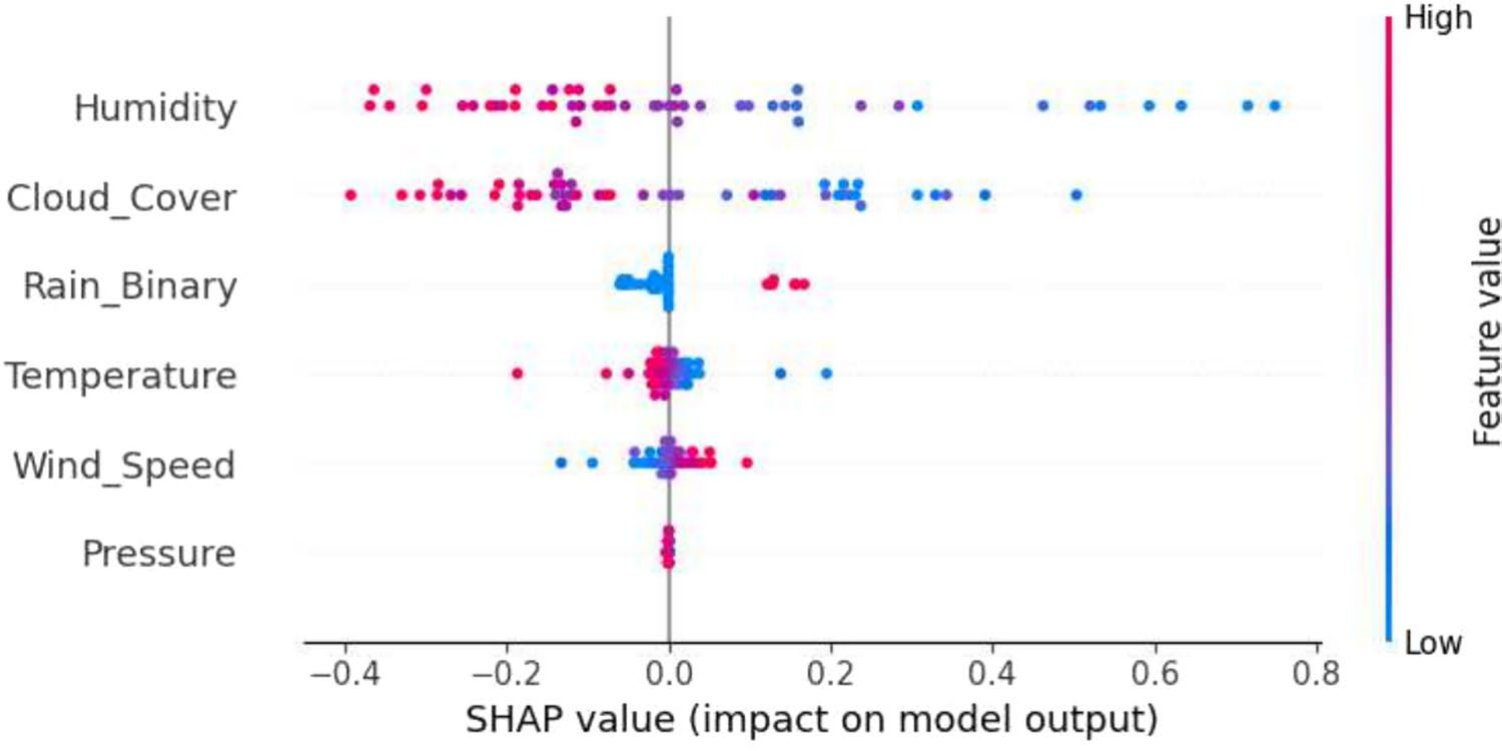
The SHAP summary plot illustrating feature contributions for class 1 (medium risk).

**Fig. 13:**
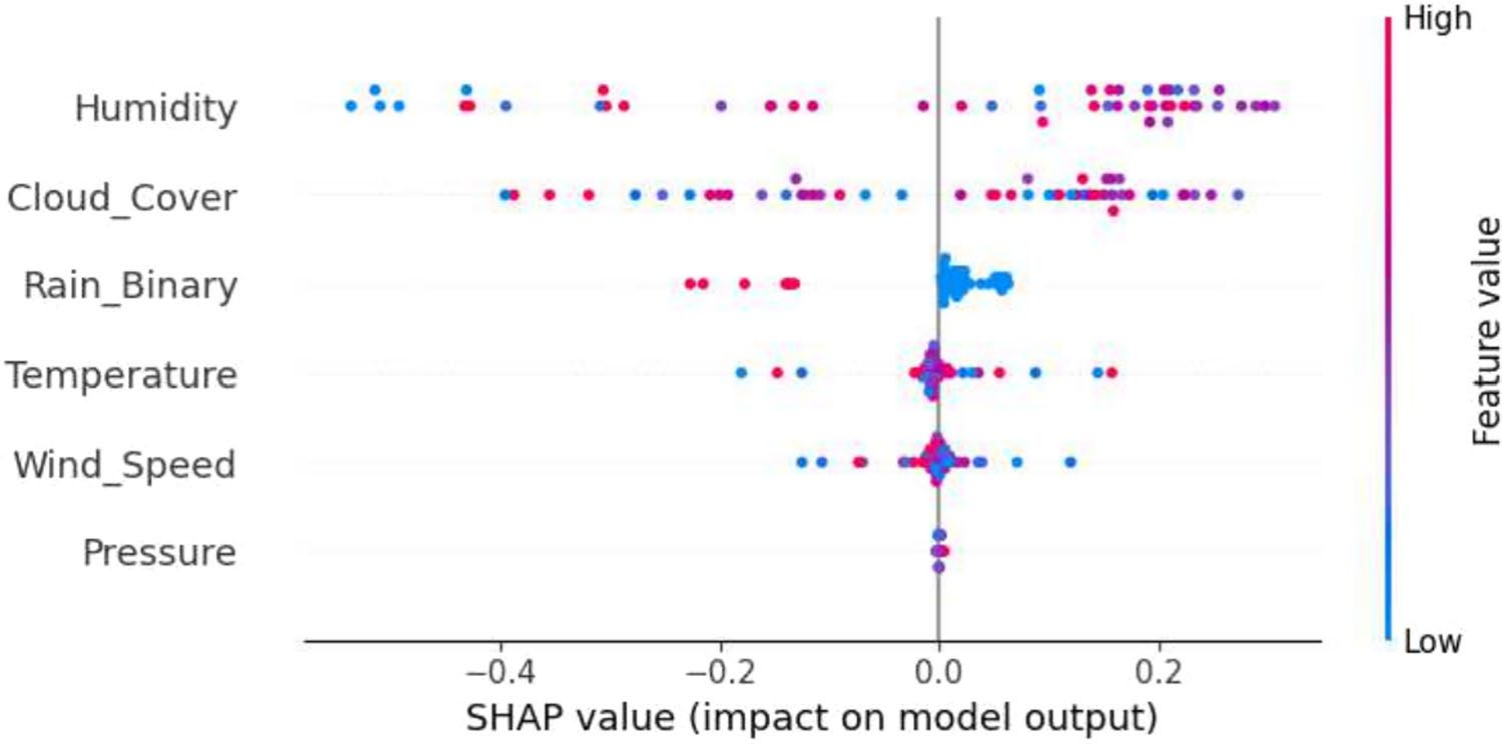
The SHAP summary plot showing key feature impacts for class 2 (high risk).

**Fig. 14:**
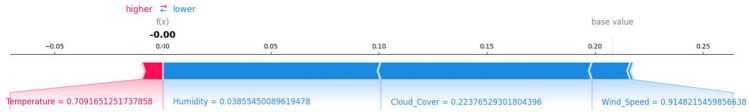
The SHAP range plot depicting the influence of various parameters on model predictions.

SHAP analysis reveals the contributions of features to classifying severity, identifying humidity, rainfall, and temperature variation as the primary predictors of disease severity. These results align with agronomic expertise, validating that the model’s decisions are based on patterns with biological significance, thereby enhancing its transparency and pragmatic utility for data-driven crop management decision-making. SHAP visualizations provided more profound insights in Figs. 12, 13, and 14.

The interpretability findings identify persistent high humidity, increased rainfall, and sudden temperature fluctuations as significant indicators of disease severity levels, which are consistent with conventional agronomic principles. This confirms the model’s accuracy as well as its explainability for effective agricultural decision support systems.

### Combined decision using late fusion

The fusion mechanism integrated predictions from both models to generate actionable outputs, such as “Predicted Disease: Late Blight—Severity: Medium.” This modular architecture ensures each model contributes independently, enhancing system scalability and reliability. Table 9 shows the prediction of disease and severity.

**Table 9:** The late fusion strategy for integrating predictions from multimodal.

Parameter	Value
Disease predicted	Late blight
Image confidence	0.35
Numeric confidence	0.65
Fused severity score	0.5340
Severity classification	Medium

### State of art comparison

The comparison of the state-of-the-art performance of our proposed research is illustrated in Table 10. We have compared it with newly published research articles from 2021 to 2025. From the analysis, it is clear that our proposed research model outperforms the state-of-the-art research, achieving higher accuracy, precision, and recall values. The analysis establishes that our research study outperforms expectations for tomato disease prediction compared to existing research.

**Table 10:** The state-of-the-art comparison table summarizing recent advances in the field.

Ref.	Year	Methodology	Model type	Accuracy
^9^	2021	Survey of deep learning techniques in agriculture	Various CNNs	91.2%
^12^	2022	CNN-based tomato leaf disease detection	CNN	90.4%
^13^	2023	LSTM for disease severity prediction using weather data	LSTM	87.3%
^14^	2024	Transformer-based multimodal fusion for early detection	Transformer	95.8%
^33^	2025	Custom CNN for real-time tomato disease detection	Custom CNN	95.2%
Proposed	2025	EfficientNetB0 with LIME + RNN with SHAP late fusion for tomato disease prediction	CNN + RNN	96.40%+99.20%

### Ablation study on model components

To validate the contribution of each modality and the efficacy of our multimodal fusion approach, we conducted an ablation study. The results, summarized in Table 11, clearly demonstrate the necessity of both components. The image-only model (EfficientNetB0) can accurately diagnose the type of disease, but does not provide information on its severity. In contrast, the weather-only model (MLP) can predict severity levels but is unable to identify the specific disease. Our proposed multimodal framework successfully provides both critical outputs simultaneously. The late fusion strategy allows each specialized model to perform its task without performance degradation, confirming the effectiveness and rationale behind our architectural design.

**Table 11:** Ablation study evaluating the contribution of each model component.

Model config.	Cls. acc.	Sev. acc.
EfficientNetB0 Only	96.40%	–
MLP Only	–	98.40%
Proposed multimodal (late fusion)	96.40%	98.40%

### Study applications

The proposed framework has significant potential for broader applications in precision agriculture beyond tomato disease diagnosis. The multimodal approach could be adapted for other crops and plant diseases, contributing to sustainable agricultural practices and food security initiatives. Additionally, the explainability components (LIME and SHAP) make the system particularly valuable for agricultural extension services and farmer education programs, helping bridge the gap between advanced AI technologies and practical farming applications.

### Study limitations

- The system is not integrated with real-time field feedback or IoT sensors, which are increasingly used in precision agriculture for context-aware decision-making.
- It uses an RNN architecture, which may not be as effective as more sophisticated ones like LSTM or GRU in identifying long temporal patterns.
- It is not yet incorporated into a mobile application, restricting farmers’ access to real-time field-level disease diagnosis.
- Although the system facilitates interpretability through LIME and SHAP, it is technical to fully comprehend the model explanations

## Conclusion

In brief, we presented a novel and explainable multimodal deep learning model that combines visual and environmental information for the classification and severity assessment of tomato leaf disease. Through the utilization of EfficientNetB0 for high-accuracy image classification and an RNN for weather-driven severity prediction, the model demonstrated excellent performance, achieving 96.7% classification accuracy and 99.20% severity estimation accuracy. The use of LIME and SHAP facilitated model interpretability by providing visual and feature-level explanations for predictions, which is essential for uptake in actual agricultural settings. Furthermore, the model demonstrates computational efficiency, with training times of under an hour for the image classifier and minutes for the severity predictor, making it a practical candidate for real-world deployment.

Our late fusion strategy enabled a modular and scalable architecture that aggregates predictions into a unified, actionable output, generating valuable knowledge for timely intervention in diseases. Our method not only enhances predictive performance but also facilitates transparency, trustworthiness, and usefulness for agricultural specialists and farmers. Although the current model demonstrates strong performance, future research will involve incorporating IoT sensor data, utilizing more sophisticated temporal models such as LSTM and GRU, and implementing a mobile-accessible interface for field-level real-time usage. This research makes a significant contribution to smart agriculture, enhancing the resistance of food systems to crop diseases at risk.

### Future work

- Integrate the real-time field data and IoT sensor inputs (like soil moisture, leaf wetness, and in-field temperature) to make the system more accurate and responsive to on-ground conditions.
- Update the RNN model to more sophisticated models, such as LSTM or GRU, to identify long-term temporal patterns of disease and weather.
- Design and deploy a web or mobile application with a user-friendly interface to allow farmers, extension officers, and agricultural field officers to access the system.

## Data Availability

The data used in this research is publicly available at https://www.kaggle.com/datasets/abdallahalidev/plantvillage-dataset and https://www.kaggle.com/datasets/zeeshier/weather-forecast-dataset
